# *“… Infections are not confined in labs…*”: Community engagement for Controlled Human Infection Studies: Opinions of researchers, bioethicists and research regulators in Uganda

**DOI:** 10.1371/journal.pone.0353964

**Published:** 2026-07-28

**Authors:** Winfred Nazziwa, Stella Neema, Alison M. Elliott, Samson B. Kaboko, Erisa Mwaka

**Affiliations:** 1 Department of Anatomy, College of Health Sciences, Makerere University, Kampala, Uganda; 2 Department of Sociology and Anthropology, College of Humanities and Social Sciences, Makerere University, Kampala, Uganda; 3 Vaccine and Research Theme, Medical Research Council/Uganda Virus Research Institute and London School of Hygiene and Tropical Medicine Uganda Research Unit, Kampala, Uganda; 4 Department of Business Administration, School of Business, Uganda Christian University, Mukono, Uganda; KEMRI-Wellcome Trust Research Programme, KENYA

## Abstract

Controlled human infection studies play a critical role in advancing our knowledge of infectious diseases and developing therapeutics. However, these studies involve knowingly exposing research participants to pathogens, raising significant ethical, social, environmental and logistical concerns. Controlled human infection studies are being conducted in settings where individuals are economically disadvantaged and may have limited access to healthcare, education, and basic social amenities, making community engagement a central element in ensuring the ethical conduct of controlled human infection studies and the protection of participants’ rights. In a cross-sectional qualitative study, we explored the views of research stakeholders on how to effectively engage local communities on controlled human infection studies. Twenty-seven key informant interviews were conducted with researchers, bioethics experts, research ethics committee members, and staff at national research regulatory bodies in Uganda between September 2023 – March 2024. A systematic inductive analysis approach was used. Due to the complexity and sensitivity of controlled human infection studies, findings reveal the necessity for early planning, adequate budgets and broad-based engagement using multiple approaches and activities. Research participants viewed the bottom-up approach as more appropriate for engaging communities. They also argued that meaningful community engagement should be a continuous process grounded in respect, justice and partnership, rather than merely a procedural or regulatory obligation. The acceptability and success of controlled human infection studies in low resource settings largely depends on effective intentional community engagement in the entire research process.

## Introduction

Health threats due to novel or re-emerging pathogens present a significant challenge to the well-being and security of people around the globe [1,2]. Responding to health threats and public health emergencies remains a priority for local governments, the World Health Organization (WHO), and other international agencies [3,4]. Efficient and timely clinical research is essential for effective responses to health threats, especially during pandemics when proven therapeutics may not be available [5,6]. Controlled human infection studies (CHIS) are among the clinical research approaches that are being adopted by sponsors and researchers because they introduce flexibility and efficiency in the research process [7–9]. These studies involve intentionally exposing research participants with pathogens under regulated environments [7,10,11]. The direct exposure of human participants to a controlled infection, enables scientists to rapidly study the progression of infection, observe the body’s immune response, develop challenge agents, and evaluate efficacy of treatments and vaccines in real time. With CHIS, results can be obtained much faster, since they require smaller sample sizes, shorter timelines while offering early efficacy signals that can inform product optimization as compared to the traditional randomized controlled trials of phase II and phase III [9,12,13]. Controlled human infection studies are a vital component of medical research [10,14,15], contributing important scientific knowledge [16–18] and accelerating vaccine development for multiple pathogens. Beyond advancing discovery, CHIS play a key role in identifying the most promising vaccine or drug candidates for further development and investment [17,19,20]. In some cases, CHIS data has supported regulatory approval of therapeutics, as demonstrated by the licensure of cholera and malaria vaccines [7,21,22]. Though, like any other research, CHIS have the potential to cause harm to individual participants and broader communities [23–26]. The harm may take various forms including but not limited to physical, social, economic, psychological and environmental [26].

As a research method, CHIS are relatively new in low-income countries (LICs) [27,28]. However, these have been used for centuries and date back to the 18th and 19^th^ centuries in high income countries [7,24]. Some of the reasons why CHIS have taken long to be implemented in LICs include the lack of research expertise, weak health systems, poor relevant infrastructure, limited funding, and lack of community understanding and reception [24,28–30]. Recently, efforts to involve research communities, foster North- South research partnerships, build infrastructure and strengthen local skills and knowledge have helped address some of the gaps, leading to a growing number of CHIS being conducted in LICs [30]. Community engagement (CE) plays a key role in the implementation and acceptance of research in LICs [31–33]. Community Engagement is “the process of working collaboratively with and through individuals and or groups of people linked by geographical location, special interests, similar situations or other individualities, to address shared challenges” [34–37]. Community engagement can also refer to an early purposive, intentional, active, and continuous collaboration between researchers and communities [38]. Other than mere participants, broader communities can be involved as partners to co-develop the research ideas, instruments and share responsibilities in conducting the research [39–41]. Community engagement supports the basic ethical principles by ensuring respect for participants and their communities, fair distribution of risks and benefits, and alignment of research with community priorities [42,43]. Community engagement helps ensure research is ethical, enhances its social value, and strengthens community ownership of the research outcomes [37,41]. Increased engagement and sustained dialogue between researchers and communities regarding CHIS can promote broader understanding, acceptance and support for CHIS [17]. This explains why CE has taken a pivotal space in medical research involving humans [37,44]. Effective community engagement not only facilitates ethical research practices but also enhances study recruitment, retention, and overall success [45]. However, there is limited consensus on how best to engage communities in the context of CHIS, particularly in LICs, where concerns about trust, exploitation, and historical inequalities are prevalent [9,46]. This study explored the views of researchers, bioethics experts and research regulators/governors on how to effectively engage local communities in the context of CHIS.

## Materials and methods

### Study design and approach

A cross-sectional exploration study was conducted among research stakeholders. Data was collected using interviews. This study was conducted within the constructivist paradigm, which seeks to interpret the world of human experience and views reality as socially constructed. Guided by this approach, data was generated through interactive dialogue between the researcher and the participants [47–50]. The design and methods used were deemed appropriate because the study aimed to gain insights into how communities should be effectively engaged in the conduct of CHIS in low resource settings.

### Study setting and participants

The study was conducted in Uganda between September 2023 – March 2024 and involved twenty-seven (27) participants drawn from three cities in central, eastern, and western parts of the country. Participants included individuals affiliated with research institutions, research ethics committees (RECs), and national regulatory bodies overseeing research. Qualitative data was collected using key informant interviews (KIIs). For this study, research stakeholders were defined as individuals who had a specific role in the oversight and conduct of research, and these included researchers, REC members, bioethics experts, and staff directly involved in research regulation at national research regulatory agencies. Hereafter, researchers, REC members, bio ethics experts, and national research regulators will collectively be referred to as research stakeholders.

### Research team and data collection

A team of five (5) conducted the KIIs, comprising of the principal investigator (NW) and four research assistants with experience in qualitative research. Before initiating any study activities, the research team underwent training on the study protocol, data collection tools, and basic research ethics to ensure they thoroughly understood and internalized all aspects of the study. Data were collected using a KII interview guide S2 Text that was adapted and developed in reference to literature on perceptions of CHIS in low resources settings [14,51]. Prior to data collection, the tool was pretested with six (6) individuals to ensure that the questions were clear, appropriate and easy to understand. Those who participated in tool pre-testing were excluded from the study. The KII interview guide consisted of unstructured questions that explored perceptions of key research stakeholders on how to effectively engage communities in the context of CHIS.

### Study procedures

The research stakeholders were individually contacted through email communication, telephone calls, and where possibly physically approached and asked for their willingness to participate in the study. For those that expressed interest in participating, an information sheet about the research and a brief write up (educational material S3 Text) containing general information about CHIS was shared through email communication a week before the interview. A follow-up phone call was made to the prospective participant a day before the interview to ascertain availability. The educational materials contained information on brief history of CHIS, what they are, what makes them different from conventional individual RCTs, countries and regions that have conducted CHIS, some of the types of infections/diseases that have used the CHIS method, the value of the CHIS, and links to video clips about CHIS. A convenient schedule (*date, time and venue)* for the interview was agreed upon with the prospective participant. Depending on the research participant preference, interviews were conducted either virtually through the zoom platform or physically at a preferred venue by the participant. The interviews were initiated and moderated by one of the research team members using a guide composed of open-ended questions. The questions encouraged easy flow of the discussions and provided participants with the opportunity to share their views on community engagement for CHIS in low resource settings. All KIIs were conducted in English, audio recorded with permission from the participants, alongside detailed note taking. On average, the KIIs lasted approximately 60–90 minutes. Interviews were conducted until the point where no new ideas were being generated from additional interviews. The transcripts were cleaned before the data analysis process.

### Ethical considerations

The study was reviewed and approved by the Makerere University School of Biomedical Sciences Research Ethics Committee (SBS- 2023−307) and the Uganda National Council for Science and Technology (SS1925ES). Written informed consent and permission to audio record was obtained from all participants prior to enrolment into the study. All participants were assured that their confidentiality would be protected, with access to personal identifying information restricted exclusively to the research team, study sponsor and authorized research regulatory bodies.

### Data analysis

The audio recorded interviews were organized and transcribed verbatim, each transcript was typed and saved using a corresponding unique identifier to the audio file. A total of twenty-seven (27) transcripts for the KIIs were analyzed continuously throughout the study using a thematic approach. The transcribed KIIs were thoroughly reviewed multiple times to internalize the content. Coding was done independently by three (3) coders, the team subsequently developed a code book S4 Text and coding framework. The coding framework was designed based on four (4) transcripts that were manually reviewed and coded to create the initial set of codes. All the transcripts were later imported into NVivo version 14 for open coding. The coding was conducted iteratively by the coders trained in qualitative data analysis. To ensure reliability, codes were repeatedly reviewed and refined by coders, leading to the development of a finalized codebook. The codes were assigned to relevant segments of the text, and related codes aggregated to form themes. Final codes and themes were organized using NVivo version 14. The themes were generated inductively, based on the patterns that emerged directly from the data. Subsequently, themes were examined for recurring patterns through iterative discussions until consensus was reached among the coders. The codes were further categorized and themes were identified. The transcripts were not returned to participants for validation; however, accuracy was ensured through repeated readings and cross- checking against the original files. The presentation of the findings adhered to the Consolidated Criteria for Reporting Qualitative Research (COREQ) checklist for qualitative studies, to enhance transparency and methodological rigor. Illustrative quotations for each emerging theme were selected for the results presentation.

## Results

### Demographic characteristics of the participants

A total of twenty-seven (27) key research stakeholders participated in the study, including sixteen (16) males and eleven (11) females. The participants were REC members (n = 9), researchers (n = 9), bioethics experts (n = 4), and officials at national bodies that are directly involved in research regulation (n = 5) as illustrated in Table 1 below.

**Table 1:** Showing demographic characteristics for research participants S1 Table.

Research participant	REC members	Bioethics experts	Researchers	National research regulators
**N = 27**	9	4	9	5
**Age range (years)**				
30–39	1		2	3
40–49	4	1	3	2
50+	4	3	4	
**Education level**				
Masters	4	3	4	4
PhD	5	1	5	1
**Gender (M/F)**	(6/3)	(3/1)	5/4	(2/3)
**Total (16/11)**				

Four (4) themes were identified from the data and these included, *(i)* Perceived relevance of CE in the conduct of CHIS, *(ii)* Planning CE for CHIS, *(iii)*Timelines for CE for CHIS and *(vi)* Approaches and activities for CE in the conduct of CHIS. The themes are presented below.

### Theme 1: Perceived relevance of community engagement in the conduct of CHIS

Participant perspectives provided insights into the necessity, significance and benefits of involving local communities in the design, plan and implementation of CHIS. Participants’ views regarding the ethical responsibility and usefulness of CE in the conduct of CHIS are illustrated below.

#### i. Community engagement as a social duty.

According to some of our research participants, CE is one way of respecting the rights and dignity of research communities. They alluded that investigators have a social duty to conduct CE for the common good. Views that one cannot conduct research without CE were expressed.

“…. *when you have been approved to do research on human participants …..you have a social duty to do CE…. for the national good, we[people] need to know…*.” **National regulator-018**.

#### ii. Raise awareness and enhance acceptability of controlled human infection studies.

The necessity to create awareness about CHIS and rapport between researchers and local communities, emerged prominently from the study findings. Participants opined that CE plays a crucial role in raising awareness about CHIS and bridging the gap between researchers and the communities they study.

“… *awareness and sensitization are key, …. not to breed mistrust……one thing that you need to do is develop sensitization and awareness materials*…”. **KII-Researcher-002**.

“*…. community engagement creates good working relationship between the researchers and the community.”* **National regulator-012**. “*….. for CHIMs [controlled human infection models] because there is a lot of lack of information related to such designs, it’s very important that we engage with the communities…*” **Researcher 015**.

In addition, CE was viewed as relevant to fostering a shared understanding of CHIS. Some participants perceived CE as a tool for sharing and verifying information to address information gaps in the community. They further noted that CE has potential to promote acceptance and reduce resistance to participation in CHIS, thus enhancing participant recruitment in CHIS.

“…. *it gives a platform for information sharing between the researcher and the community…. it also improves the informed consent process…,* “… *it is one of those vital tools in research, it improves the acceptability of the study [CHI] at a community level* …*””* **National regulator-012**. “…. *and you will have an increase on the recruitment rate … you need the community to do that because they understand the study [CHI] better…*” **Researcher-023**.

#### iii. Build community trust and inform decision making.

Participants also noted that transparent communication, with clear explanations of CHIS objectives and safety assurances, is essential for allaying fears, dispelling misinformation and responding to community concerns early in the study. Especially since CHIS is a relatively new research approach in LICs like Uganda.

*“…if they bring a CHI study to Africa, and the PI is from the West …rumors will say that they are trying to infect us...I think initially, we should be trying to address those fears through engagements…”* **Bioethics expert-007**.

“*…if you don’t engage early, you just come in maybe a few days before enrollment, the community will be very apprehensive about what you’re telling then. CE gives them time to inquire from other alternative sources of information, verify that this thing [CHIS] is a safe* *and then they make up their mind…”* **REC member-008**.

#### iv. Identifying and mitigating risks.

Participants perceived CE as instrumental in shaping study procedures, identifying and minimizing risk in CHIS. Beyond physical risks, participants noted that CHIS may pose social risks, such as stigma or family disruptions. Community engagement may help anticipate these risks and co-develop mitigation strategies.

“…*. CE is a crucial part of timely risk mitigation…*.” **National regulator-011**. “…. *You will not have your CHI participants having stigma. You will not have them calling other people names who have been part of the study [CHI] … you need the community to do that because they understand the study [CHI] better…*” **Researcher-023**. “….*……*. *able to address social risks……so, there is a need for extensive community engagement for the communities to be informed about this [CHIS]*…” **National regulator-017**.

### Theme 2: Planning community engagement for controlled human infection studies

Planning for CE, particularly for CHIS, is important and preparations require a carefully structured approach as narrated by the participants. “….. *ideally, each study should have a CE plan and sensitization mechanism…*”. **Researcher-002,** “…. *for these protocols [CHIS], I know this is a requirement, the CE plan…*”. **REC member-026**.

#### i. Development of community engagement strategy.

Study participants advised that research teams need to proactively plan for CE due to the design of CHIS. They proposed development of a comprehensive and well-structured CE strategy, aligned with the national ethical frameworks and approved by the research ethics committee.

“… *I really think such a study [CHI] would be required to have a comprehensive community engagement strategy ….”* **Bioethics expert-024**.

“…. *of course it has to be structured, purposeful, objective and kind of responsive and feedback driven. …and it has to be in an approved kind of manner….”*. **National regulator-018**.

Furthermore, participants noted that researchers should not merely develop CE plans for CHIS protocols, rather the plans ought to be meaningfully implemented. Also, during the design of these strategies, a bottom-up approach was viewed as the most appropriate and effective means for ensuring their relevance and impact.

“…. *CHIM studies, CE is of utmost importance, and it’s imperative to ensure that the CE plan is not only created but also diligently implemented and followed through…..”* **National Regulator-011**.

“…. *very effective is the bottom-up approach and this involves starting with the smallest unit of that community…….*.” **REC-Member-010**.

#### ii. Allocation of adequate resources for community engagement.

One participant highlighted that adequate funding should be planned and ensured, and researchers ought to take this into account when designing/ planning CE activities for CHIS. *“… community engagement is now a component of the budget. You have to budget good money for CE*…” **Researcher-015.**

### Theme 3: Timelines for community engagement for controlled human infection studies

Participants opined that appropriate timing of CE more so in a low resource setting ought to be early and ongoing through out the lifecycle of the research. ……*CE should start as early as possible. …*” **REC member-013**, “…. *right from the beginning….*” **Research regulator-018**… *“… at various stages … would be great because the consulting process is really there to also guide the researcher…*” **REC member-019**.

#### i. Initiate planning for community engagement at study ideation.

Participants expressed diverse views on the appropriate timing of CE, with many advocating for early involvement of research communities during the study conception stage. They emphasized that initiating CE at the earliest opportunity is crucial for ensuring that CHIS are culturally appropriate and ethically sound.

“.. *I think once they have conceived the idea, that’s when the engagement should start.*...” **REC member-027**. “*… I think for these studies [CHIS] once you’ve thought about the study, start engaging and have everyone on board….”* **REC-member-004**.

Given the nature and design of CHIS, participants further noted that CE should be a continuous process rather than a one-time consultation or activity, to ensure that community views are addressed at every stage of the research process.

**“***… there should be continuous engagement to ensure that the community’s views are taken care of at every stage in the study…*” **Researcher-023**.

*“… study engagement should not be done at once, should be continuous…. not a one-time thing….”* **REC member −003**. *“… CE is not a one-off* …” **National regulator-012**

#### ii. Phases for community engagement.

From participants’ voices, CE may be done in a phased approach. The first phase occurs during protocol design and development, followed by engagement during ethical and regulatory reviews. With participants noting that CE should take place prior to submitting protocols to the REC and other regulatory bodies.

“…. *I think… it has to happen before … approvals [REC, UNCST, NDA]* …” **REC member-004**. “…. *the engagements... before we submit the protocols to the IRB, it’s continuous, because someone can make a very important recommendation that you need to incorporate in the design….*” **Researcher-015**.

“*…. right from the design of the study, when the researcher has an idea in mind….… from the design of the protocol and the methods because when you’re designing the protocol there may be some community-specific characteristics actually that you may benefit from if you engage them from the beginning*...” **Researcher-023**.

The third phase encompasses recruitment, enrolment, and implementation, with a final phase addressing post-study activities such as dissemination. CE was considered essential during recruitment and enrolment and should be sustained throughout implementation to address any emerging concerns.

“…. *At the consenting time, at the recruitment points and at dissemination….*” **REC member-010**. “…. *even during the study there should be continuous engagement with the members of the leadership, in the community, and others…*.” **REC member-008**.

“…. *CE should commence during the study design phase. Engaging communities and stakeholders at this early stage helps you understand their perspectives on the work you intend to undertake and identify any misconceptions….”.* **National regulator-011**.

Although, majority of our study participants advocated for early CE as illustrated above, one participant noted that currently, researchers engage communities later after protocol approvals. “…. *But what people do with CE…. they first develop the protocols, even have them approved by REC. Then they come and start doing CE with things that will not work…*.” **REC member-013**.

### Theme 4: Approaches and activities for community engagement in conduct of CHIS

Given that CHIS involves scientific complexities as well as unique ethical and social considerations, participants opined that multiple approaches are required to effectively engage diverse audiences, including potential participants, their families, leaders, and the public. Participants noted that a single method cannot adequately address the varying levels of understanding, cultural context and access to information.

“….. *I’m saying you need multiple approaches. One may not be enough, to get the different people and concerns*…..” **REC member-003**. *“…multiple approaches where you can engage different people, different groups…one way, one method may not be enough….”* **REC member-019**.

Participants identified a few approaches including initial or formative consultations, leveraging community structures and groups, community leaders, community events, mass media, community advisory boards (CABs), and national engagement. These approaches are expounded below.

#### i. Formative consultations.

Initial consultations with the research communities are essential, study participants cited mapping of the research area and population through formative discussions as an approach for CE. Area and stakeholder mapping helps to identify key stakeholders within the communities, plan resources and the appropriate engagement activities that can be conducted among the population.

“ …. *first kind of get some formatting work to give information on how best, what model of engagement would people best understand…”.* **Researcher-009**. “… *do a mapping to* *understand how to engage …*” **National regulator-018**.

#### ii. Existing community structures and stakeholders.

Studying and understanding the administrative structures of the given community is important in any research process. Community structures could be formal and informal systems of organisation and influence within a community. Participants highlighted some existing community structures and stakeholders that can be used for CE. For instance, district health officials (DHO), ministry of health officials (MoH), and other health professionals at district and village level such as village health committees and other local structures. Understanding the organizational structure of the area under study was emphasized.

*“…..if you’re going to get the girls, let’s say from Katanga or Bwaise [suburbs in Kampala], you have to study the organizational structure of where that girl comes from*. …. *you study the environment and know who is influential and what is the organizational structure of the population you are recruiting the participants from*….” **Researcher-015**.

“…*if you are going to conduct a CHI study and do not engage the MoH, the district officers to have them understand what you are going to do, you will not get buy-in from the community. They will actually discourage the community because they are the influencers, they are the movers of things….*” **National Regulator 018**.

“… *deal with the VHTs and the health committees at the lowest levels around health centers to mobilize, … that’s if you’re in rural Uganda…..”* **Bioethics expert −024***.*

#### iii. National engagements for controlled human infection studies.

In addition, study findings reveal diverse perceptions of stakeholders who should be engaged in CHIS particularly in low-resource settings. Participants mentioned a wide and national engagement that brings on board different groups of stakeholders in the community, this was mentioned because of the complexity of CHIS. Four participants opined the need to have a national engagement for CHIS that involves everybody. One participant cited that the entire community should be engaged since infections are not confined to the laboratory. In conducting a national engagement, one participant proposed a phased approach.

“… *I think all stakeholders*…… *personally I would call for a national engagement strategy because in the end, this affects different groups of people …*…. *this has a national picture but a phased approach of engagement*…*”* **National regulator-018**.

*“…infections affect communities on a public health model…the representatives of the entire community should be engaged. Because infections are not confined in labs. They move out into the rest of the community…*”. **Researcher-002**.

“…. *everyone, I would think, because it is a very scientific and technical field, you’d have to start with the scientists, the health professionals, … even amongst us the regulators, …… we are hesitant really to accept the design [CHIS]….”* **National Regulator-024**.

Our research participants mentioned a few of the specific groups of individuals that should be engaged, including the target population where potential participants will be drawn from. researchers, research regulators and all stakeholders. Due to the potential for social, environmental and third-party risks related to CHIS, participants mentioned that individuals connected with the potential participants should be engaged as well.

“….*so, it is really the community where you’re going to get the participants, it is at the villages where the participants return. … that is where they live, the neighborhoods that are at risk [third party risks] ….”*. **REC member −013**.

“… it *should be all those people associated with the potential participants. Even if they are not part of the inclusion criteria, because there is a potential for infection [third party risks], there is a potential for social connection with volunteers...”* **REC member-003**.

*“…. rather than coming to REC and UNCST [national research regulatory body] with your full proposal, I think it is to be very beneficial or makes work easy for the researcher to engage the regulators [REC, UNCST, NDA] in the process of developing this idea….*.” **REC member-019.**

Finally, the private sector is hardly considered for inclusion in research activities. One study participant mentioned that engaging the private sector is important. “…. *It’s also essential to involve the private sector, in Uganda, if CHIMs yield promising results that can be turned into innovations to enhance the quality of life for Ugandans, the private sector should understand and be involved. Otherwise, you might have valuable results but lack the capacity to implement interventions for the benefit of the communities..*.” **National regulator −011**.

#### iv. Community leaders.

Among the primary groups identified for engagement were community leaders including traditional, political, cultural, religious, opinion leaders and respected/influential community members. Participants underscored the importance of prioritizing community leaders for CE because of the trust they command and the influence they wield. Political leaders and legislative bodies were also recognized as important stakeholders to involve to seek their views and secure buy in for CHIS. Overall, community leaders were viewed as gatekeepers to research communities.

“…. *the critical stakeholders and gatekeepers to the community…. the local leaders, for example, LC 1 persons…”.* **Bioethics expert-024**.

“*……leaders are actually key and good for stakeholder engagement, … religious leaders, cultural leaders because they help to demystify any myths that maybe associated to CHIS, they are the ears on the ground in the community….”* **Researcher-006**.

*“…. It [CHIS] should go to parliament. let’s see how the members of parliament perceive it...”* **REC member-001**.

#### v. Community events.

Study participants opined that community-based events are necessary for CE for CHIS. Community based events such as community meetings (village, parish meeting), workshops, seminars, sports events, edutainment (drama skits) and community health outreach were highlighted by the study participants. They shared experiences regarding community meetings. *“…. community events can be very useful; you can meet them at different community events….*” **REC member-008**. “…. *We have taken advantage of village meetings and then you request that at the end of the meeting, you have something to say. They give you some minutes, and you talk about the study and of course you facilitate the meeting*…” **Researcher-015**.

Some participants cited workshops, seminars and meetings, in which leaders and influential/respected individuals in the community are part. Such meetings are crucial for seeking community views about the different CHIS procedures.

“*….. town hall meetings, village meetings to explain to people and get their views and opinions …around various parts of CHI studies such as confinement* …... *call meetings and discuss about the study when all the influential people are there*….” **REC member-026**.

“*….. direct engagement in terms of workshops with members like…. Leadership at local council level, (chair, secretary for health, secretary for defense, people who are influential in the community …”* **REC-member-008**.

Also, one participant noted that engaging communities through village or community meetings may aid in identifying potential CAB members that can be appointed to serve on the CAB. “…. *And then if the area doesn’t have a CAB, from those meetings, that’s where, you see the active people, …. and make them your CAB members*…” **Researcher-015**. Community advisory boards are one of the approaches for CE.

Moreso, edutainment and or drama skits were viewed as vital tools for sharing information with community members. While also proposing simulation or dry runs of CHIS procedures to aid in identifying potential challenges and refine the protocol before the actual study begins. Sports events, particularly football matches, were highlighted as a key gathering that attracts large audiences and provides opportunities for researchers to sensitize diverse groups and gather real-time feedback. Additionally, participants pointed to community health outreach activities such as providing healthcare services and health talks as vital events for creating awareness about CHIS.

“… *giving information through … plays that are inform of role-playing or maybe mimicking, what would happen in the CHI research so that people can easily understand the information that you are trying to pass on*…” **Researcher-009**.

“*…do a dry run within the community to test their knowledge; what do they know about CHIM, any prior knowledge? …. This can help design a better engagement strategy with the communities…*…*you can also organize maybe a football match on a Sunday and educate them about CHIM… tell them about their contributions, how they can benefit from that….”.* **National regulatol-012**. “……*while you are there*, *do screening and treatment for anything you can*…*we normally call them health talks…”* **Researcher- 006**.

#### vi. Community advisory boards.

The other engagement approach that was mentioned is the CAB, which was perceived as a key strategy for CE for CHIS. Study participants acknowledged the value of CABs as a group of people who facilitate dialogue between the research team and the community. They noted that CAB members are identified from the community and are familiar with the community processes. In addition, for CHIS, participants recommended that CABs should be considered as one of the mandatory approaches for engaging communities. This is due to the design of CHIS that is likely to raise ethical concerns.

“…. *community advisory boards are one of the strategies that should be employed for engaging the community……. these boards are highly significant because they serve as a bridge between the study team and potential volunteers, often originating from the community itself. They are attuned to what’s happening in the community, even things that researchers might not be aware of….”* **National regulator-011**.

“… *In CHIMs, CABs should be considered a mandatory component of CE..*..... *CABs are essential because they represent community interests and are diligent in ensuring the well-being and safety of study participants….*” **National regulator-011**.

The CAB was viewed as an approach that can help research teams navigate concerns more responsibly and ensure that CHIS is conducted with respect. Our study participants opined that CAB members may participate in the development of recruitment materials such informed consent documents, educational resources and any other data collections tools. However, advised engaging the CAB at the initial stages of protocol design.

*“…. they [CAB] participate in the translation of some of the documents, like the videos or translation of the informed consent forms to local languages……… They must be engaged, and the engagement is not giving them information about the design or the protocol. It is engagement starting from the beginning of the design of the protocol. So that they participate in providing feedback on the method of recruitment of potential participants*….” **Resaercher-009**.

One of the participants acknowledged the important role that CABs may play in CE for CHIS, although suggested that CAB should be used alongside other approaches. From the participant’s perspective, CABs are necessary but not sufficient for engaging local communities on CHIS.

*“…. so, to me, CABs are necessary, but they have to be carefully selected...It’s a good approach, simply a component of CE. But researchers should go beyond community advisory boards and do other activities that make the community accept CHI research….”.* **Bioethics expert −007**.

#### vii. Mass media.

Mass media was mentioned by study participants as an approach for CE. Participants accentuated the importance of mass media for CE and its wide coverage as a vantage over other engagement approaches. Some of the mass media outlets mentioned encompassed broadcast media like radio and television, through talks show or dialogues, community megaphones or loudspeakers and community public address system drives.

“… *involve the media because the media can take it [CHIS messages] beyond a workshop by sharing through different media channels like TVs, radios and so on….”* **National regulator-012**.

“…… *I hear people going around on microphones with speakers, …. they go around, ….*” **Researcher-006**. “… *village public address systems and community drives… ones of walking/driving around… the message should be consistent and well packaged….”* **REC member-005**.

Nonetheless, some participants cautioned that mass media should be engaged skillfully because the likelihood of distorting messages is high. There is need to control the information that is going out. Participants suggested provision of talking points as the mechanisms of ensuring that messages are not distorted and assigning research team members to work with the media professionals.

“….*, media is one of those key stakeholders and they help in disseminating but you need to control them so that they disseminate the right information. They can distort messages, and it* *renders the whole thing useless* …” **National regulator-012**.

“….. *haaaa… media is special. Hahahaaa… they can turn the ball right side up, to be polite…the packaging for media has to be very intentional*…. *for media, give them talking points, because media has a way in which it can turn the interpretation or everything around. and even if something was meant to be good, it can be turned the other way. …packaging is important,* …...*also have the communication strategist [media person] work with the research team and make sure that the packaging is right, make sure that the translation is good….*” **REC member-005**.

Furthermore, participants suggested using other media platforms in addition to the conventional mass media such as TV and radio. However, cautioned that, the choice of media should also be based on the target audience.

“…. *I would not ignore the digital platforms as well. I will not just use only the conventional media like TV, radio and all that, I would even use the nonconventional ones…”.* **KII-REC-member-003**.

“…. *Try and see the population being targeted then tailor whatever media messages, …..I would not be fixated like on a certain line that it must be radios, unless most of my target people are going to be on radios….”* **REC member-005**.

## Discussion

Globally, respecting and protecting research communities is an ethical obligation [34,52,53]. Researchers and sponsors are responsible for engaging community stakeholders in the entire research process, from ideation and protocol design to implementation and the post study phase [31,34,38]. Such engagements form the foundation of ethical research, particularly in the context of CHIS, which may often be met with skepticism due to their perceived risks and unfamiliar nature [30,54]. Although CE is an indispensable aspect of any research [34,55] more so CHIS, there is sparsely published literature on how investigators can effectively engage research communities on CHIS. Our findings reveal the necessity for early planning, adequate budgets and broad-based engagement using multiple approaches and activities. Study participants viewed the bottom-up approach for involvement as more suitable. Also, well-resourced CE efforts that are continuous were viewed as central in building community trust, improving study design, and enhancing ethical acceptability of CHIS in LICs. Participants’ views provide valuable insights into the benefits associated with involving local communities in the conceptualization, plan, design, and implementation of CHIS.

Community engagement is one way of respecting research communities, it allows community members to be informed and involved in the decision-making process for research [31,41]. Meaningful CE can be one tactic to addressing particular issues that CHIS raise particularly in local communities, where views may inform the conduct of CHIS [30]. Such engagement can enlighten the ethical conduct and acceptability of CHIS in LICs. Also, CE provides opportunities for sharing information, listening to, and responding to concerns from potential participants and the larger community in which a CHI study would be conducted [9]. Engagement was regarded as a tool that augments trust and endorsement. Such alignment can only be facilitated through active and intentional CE [46]. Involving the community in the research process increases awareness about CHIS and address fears, rumors and any concerns early on [30]. Thus, boosting acceptability, participation, practicability, pragmatic value and significance of the CHI design, implementation, and outcome dissemination [31,45].

The CHIS method is complex, sensitive, contentious, and counter intuitive. In LICs with limited resources, the planning and timing for CE particularly for CHIS is paramount. While having a CE plan is a regulatory requirement for research in Uganda [34,56], planning for CE should not be just a ritual step. Preparing for CE should be an introductory strategy that supports ethical and effective research. Arranging CE for CHIS requires intentional, early and ethically grounded steps to ensure trust, mutual understanding, and responsible research conduct [56,57]. The process of planning for meaningful CE is most effective if it is not treated as an afterthought but as an integral component of CHI study. The preparations require a carefully structured and culturally sensitive approach. Our study participants recommended development of a comprehensive CE strategy that is co-created with the local community members. It is essential to have an all-inclusive CE strategy that is well structured, with clear objectives, goals, target audience and feedback oriented [58]. In many research projects CE is treated as a secondary activity and underfunded [31,33,59,60]. Study findings reveal the necessity to allocate adequate funds for CE activates, as an enabler for planning and effective implementation of CE. Well-resourced CE strategies are more likely to enhance diverse reach and quality community involvement [41,56,61]. Without sufficient financial provisions for CE, efforts to foster trust and collaboration between researchers and communities are significantly undermined. Arranging for CE, adequate financial commitment by the sponsor and funder are essential for ethically sound CHIS.

In addition, preparation for CE for CHIS in LICs requires a bottom-up engagement approach. Researchers need to leverage the community entry points such as local leaders, existing community structures and key stakeholders. These ought to be engaged in the co-creation of messages and consultation structures [57,62,63]. Study participants advocated for a more inclusive and culturally appropriate dialogue. This approach contrasts with the usual top-down information sharing models, which often fail to address local concerns [57,64,65]. In LICs the bottom-up approach may ease community ownership of CHIS, align scientific goals with community needs, build trust and smoothen recruitment of potential participants, hence improve CHI research outcomes in LICs. Conducting CHIS in LICs adds additional ethical, social, scientific, environmental and logistical layers that require early, careful and phased approach CE. The appropriate timing for CE more so in a low resource setting county ought to be early, ongoing and adaptive through out the CHIS lifecycle. Early planning allows for inclusion of community views in deciding about communication strategies, risk categorization and participant support mechanisms. This aligns with other scholars who recommend proactive CE in research planning, specifically for study methods that are complex, not easy to comprehend, involve heightened risks not only to individual participants but also the environment and wider research community, as is the case with CHIS [33,66].

Findings suggest that effective CE for CHIS requires a multi-pronged flexible strategy that leverages local knowledge and structures to ensure inclusivity and transparency. A range of approaches and activities can be undertaken to achieve the goals of CHI research in LICs [67]. These may include formative consultations, community events, mass media, existing community structures, community leaders and the CAB [62]. Formative consultations also referred to as “affective” engagement [64] can be used in the initial stages of CHIS planning; this approach is a basis for developing effective CE strategies/plans. It aids the research team to gain an informed understanding of local populations characteristics, needs, socio-cultural norms and practices, governance structures, power dynamics, perceptions, and local history of research generally [63]. In the context of CHIS, formative consultations may enable researchers to understand local perceptions of human intentional infection, identify community concerns and adopt appropriate engagement strategies. Early community involvement aligns with established international and national ethical frameworks which emphasize that CE should begin well before participants recruitment and continue throughout the research life cycle [34,36,56].

Effective CE may require working through community structures, and with leaders or stakeholders that have sufficient understanding of the target community depending on CHIS CE goals [30]. Some of the community structures include but are not limited to local government technical departments such as (District Education Department, local administrative structures, District Health Teams, School Management Committees, Gender Culture and Community Development Department), village health teams, civil society organizations, community-based organizations such as farmers’ associations, youth groups, patient groups, peer-led groups, women groups, business associations, community health workers and health care providers [34,63,68]. In LICs, research communities have several leaders that can play a role in CE. They have considerable authority and influence on community opinion by virtue of their roles, status, knowledge, reputation, or other attributes. Dialogue with these leaders can establish endearing relationships built on trust, transparency, and commitment to research activities. The leaders and stakeholders do not only act as gatekeepers but also as cultural advisors who can construe and relay key CHI study messages to the target communities [64,68]. For effective CE researchers need identify the most appropriate existing community structure or stakeholder to facilitate community entry, consultation, and involvement. However, researchers need to be cognizant of the fact that use of community leaders/exiting structures as an approach for CE may raise questions about inclusion, as traditional structures may not always represent the full diversity of community perspectives [68–70]. Also, ensuring that marginalized and dissenting voices are heard remains a key challenge.

Community events are key engagement platforms in LICs where formal systems may be weak, literacy levels vary, and personal interactions remain as the most effective channel for communication and partnership. Community events, for instance cultural gatherings, market days, seminars, edutainment and drama skits, community meetings, sports events, community health outreach activities and public meetings [60]. Community-based activities offer informal yet powerful spaces for engagement since they can attract a large number of community members [67]. These events not only help explain the science behind CHIS in comprehensible languages but also create opportunities for researchers to listen to concerns and respond transparently [30]. Such interactive formats enhance community understanding and contribute to the perception of the research as a collaborative rather than an extractive process. Despite the strengths of this approach, several challenges may arise, for instance high mobility of the population, lack of unified community leadership may complicate the engagement efforts. The intensity and resource demands of sustained CE events underline the need for adequate funding and institutional commitment [60].

Community Advisory Boards (CAB) also referred to as community advisory groups (CAGs), can be an essential tool for responsive, ethical and community centered engagement for CHIS [30]. Our study findings are consistent with earlier research, which similarly demonstrated/identifies CAB as a vital link, that facilitates dialogue between the research community and the research team [71,72]. Their formation and involvement can play an important role in establishing two-way communication between researchers and communities. The CABs facilitate the flow of information; help identify potential ethical or logistical issues and support culturally sensitive decision making [73]. In global health research CABs are increasingly recognized as a valuable mechanism for community input and oversight [74]. However, the capacity of the CABs and various challenges that affect their functionality in resource limited settings should be considered and addressed for effective engagement of communities during the conduct of CHIS [72,74].

The use of mass media as an approach for CE is another significant tool for expanding outreach. It may be preferable when reaching out to a broader community, non-technical audiences and where the research is not under one geographical area [75]. Mass media can be used for education, sensitization, communication as well as a participant recruitment strategy [76–78]. Some of the mass media channels include but are not limited to: print media (newspapers, flyers, brochures, posters), broadcast media (radio, television, films), digital media (internet and mobile telephones for phone text messages/messaging, calls), community megaphones or loudspeakers, community public address system drives and Internet media (social media, emails, websites) [75]. Although, its use may require careful management to avoid sensationalism [75] and ensure the accurate presentation of CHI study objectives and risks [79]. To minimize exaggeration, CHIS information to the media outlets should be accurate, thoughtful and tailored to the type of media channel [76,79]. In addition, working with social scientists and communication experts is advisable to enable present information in a way that is acceptable to the communities [30,80,81].

Due to the complexity and scientific nature of CHIS design, our participants opined engaging various stakeholders in the community. Broad based engagement guarantees that the CHI research is ethically sound and locally acceptable. Although only a few individuals are enrolled in research, everyone is part of the social environment in which the CHI study is likely to be conducted. To fully engage the community, some scholars/authors assert the need to involve the microlevel, meso level and macrolevel stakeholders [82]. These may include but not limited to broad research community, potential study participants and their close family members, research regulators, community leaders, policy makers, health care providers and public health officials [82].

## Conclusion

The success of CHIS, especially in contexts where research mistrust and health inequalities are prevalent depends heavily on the quality and authenticity of CE. The highlighted CE approaches and activities provide guidance for designing and implementing CE for CHIS in LICs. We assert that CE should not be a peripheral activity, but a core driver of CHI research, social accountability, and long-term impact. For CE to be effective, it should not be treated as a checkbox but as a continuous ethical process grounded in respect, reciprocity and responsiveness of the local community. Therefore, understanding the dynamics of CE in CHIS is essential for designing a more effective, inclusive, and trustworthy research not only in Uganda but also similar settings around the world. In addition, it is essential to build local capacity for CE planning and implementation, particularly in contexts where CHIS is relatively new or ethically contentious. If tailored to context and supported by genuine partnerships, CE may enhance the ethical rigor, scientific quality and social value of CHI research in low resource settings.

### Limitations

Since, CHIS is one of the uncommon research methods in Uganda, majority of our study participants were initially unfamiliar with the concept of intentional human infection. To address this, educational materials containing general information about CHIS were shared via email prior to the interviews. This enabled participants to engage more knowledgeably and promoted collection of valuable contextual data. Nonetheless, the provision of information may have influenced the discussion and study results towards desired outcomes of the research. While our findings have important implications for designing CE in future CHIS and other complex or contentious clinical research, results are limited to views of selected research stakeholders. The views mainly pertain to the relevance of CE, approaches and activities for CE, Timing for CE and crucial community members to be engaged in LICs. Future research should focus on systematically evaluating the impact of the different CE approaches and methods on community trust, participant recruitment and study outcomes.

## Supporting information

S1 Table Research Participant demographics. (DOCX)

S2 Text Key informant interview guide. (DOCX)

S3 Text Educational material – KIIs. (DOCX)

S4 Text Code book. (DOCX)
