# Supplementary material for: “… Infections are not confined in labs…”: Community engagement for Controlled Human Infection Studies: Opinions of researchers, bioethicists and research regulators in Uganda
Source: PLoS One. 2026 Jul 28;21(7):e0353964. doi: 10.1371/journal.pone.0353964 (PMC13411932; doi:10.1371/journal.pone.0353964)
Supplement: S1 Table — (DOCX) [file pone.0353964.s001.docx]

**S1 Table. Research Participant Demographics**

| **Research participant**  **N=27** | **REC members** | **Bioethics experts** | **Researchers** | **National research regulators** |
| --- | --- | --- | --- | --- |
|  | 9 | 4 | 9 | 5 |
| **Age range (years)**  30 – 39  40 – 49  50+ |  |  |  |  |
|  | 1 |  | 2 | 3 |
|  | 4 | 1 | 3 | 2 |
|  | 4 | 3 | 4 |  |
| **Education level**  Masters | 4 | 3 | 4 | 4 |
| PhD | 5 | 1 | 5 | 1 |
| **Gender (M/F)** | (6/3) | (3/1) | 5/4 | (2/3) |
| **Total (16/11)** | | | | |
