# Supplementary material for: “… Infections are not confined in labs…”: Community engagement for Controlled Human Infection Studies: Opinions of researchers, bioethicists and research regulators in Uganda
Source: PLoS One. 2026 Jul 28;21(7):e0353964. doi: 10.1371/journal.pone.0353964 (PMC13411932; doi:10.1371/journal.pone.0353964)
Supplement: S2 Text — (DOCX) [file pone.0353964.s002.docx]

**KEY INFORMANT INTERVIEW GUIDE FOR REC MEMBERS, NATIONAL REGULATORS, BIOETHICS EXPERTS AND RESEARCHERS**

**Participant details:**

1. Participant ID: _____________

2. Age in years: _______ (20 -30, 40 – 50, 50 – 60, 70 above)- (codes for age range)

3. Sex: Male/ Female

4. Education: Undergraduate/Graduate/Postgraduate/Doctorate

5. Specify the highest degree: ________________________

6. Role in the REC/bioethics experts/national regulator/: Basic Chairperson/Medical Scientist/ Legal Expert/Social Scientist/Lay person/Clinician/Others

7. Others (Explain) ________________________

8. Years of experience as REC member/staff at regulatory agency/bioethics expert/Researcher

**Community Engagement for controlled Human infection studies (CHIS)**

1. Is community engagement important to CHIS?
2. Who is the community that should be engaged in the conduct of controlled human infection studies?
3. How should that community mentioned above be engaged in the conduct of controlled human infection studies?
4. When should the community engagement for CHIS be done?
5. Who should conduct community engagement for CHIS?
6. How can the sensitization of media and policymakers be done if one is to conduct a CHIS in Uganda?
7. In your opinion, what community engagement activities/strategies should be conducted before, during and after the conduct of CHIS? Probes: Media campaigns? Community Advisory Boards? Community structures and stakeholders? Involvement of government partners?
8. How can transparent and continuing communications or engagement be implemented during the conduct of CHIS
9. How can social media be involved in a positive way in engaging communities on CHIS?
10. In your opinion what are the other CE strategies and activities that can be used in the conduct of CHIS?

Do you have any other concerns or comments or anything that you would like to share? Please share your thoughts.

**THANK YOU**
