# Supplementary material for: “… Infections are not confined in labs…”: Community engagement for Controlled Human Infection Studies: Opinions of researchers, bioethicists and research regulators in Uganda
Source: PLoS One. 2026 Jul 28;21(7):e0353964. doi: 10.1371/journal.pone.0353964 (PMC13411932; doi:10.1371/journal.pone.0353964)
Supplement: S3 Text — (DOCX) [file pone.0353964.s003.docx]

**BRIEF INFORMATION ABOUT CONTROLLED HUMAN INFECTION STUDIES**

**Introduction**

In clinical research, the Randomized Controlled Trial (RCT) is known as the most scientifically rigorous method of hypothesis testing and has traditionally been the gold standard for evaluating the efficacy and safety of new interventions. However, due to the methodological challenges posed by individual RCTs *(require large sample sizes, long duration/time consuming, high costs*) clinical research sponsors and or investigators are increasingly adopting modifications to conventional trial designs and methodologies that introduce flexibility and efficiency. One of the approaches being adopted is the Controlled Human Infection Studies.

**What Are Controlled Human Infection Studies**

Controlled Human Infection Studies (CHIs) have a diverse nomenclature. They are also known as, Controlled Human Infection Models (CHIMs), Microbial Challenge Studies (MCS), Human Infection Challenge (HIC) studies, Human Challenge Studies (HCS), Human Challenge Trials (HCT), Human Challenge Models (HCM), Volunteer Infection Studies (VIS) and Challenge studies (CS).

Controlled human infection study (CHIs) is a clinical research method that involves *intentionally infecting*/giving healthy research participants *pathogens/germs* (or other micro-organisms) with the primary aims of

1. testing vaccines or therapeutics these could be new or proven therapeutics,
2. generating knowledge regarding the natural history of infectious diseases,
3. developing “models of infection” of infecting participants with a particular micro-organism). Models of infection are reliable methods to be used in studies with aims “a or b” above.

These studies are called “controlled” because they involve *controlling/regulating* the selection and production of the *models of infection/strains*, the *route of administration*, the *timing, and dose of infection*. Also infection is in a *controlled* environment; infection with micro-organisms causing no disease or disease that is self-limiting or can be *controlled* with early diagnosis or effective cures/treatments; or *controlling* who is being infected (subjected to other experimental interventions). In some cases, research participants that have been infected are retained under controlled clinical conditions in a *residential facility*, where they can be carefully monitored for days/weeks/months *(depending on the nature of infection/disease under study)* until they are no longer infectious.

It is important to note that research participants in CHIs are infected with pathogens for diseases that are either *self- limiting or fully treatable.* This is with the exception of the severe acute respiratory syndrome coronavirus 2 (SARS-CoV-2), where CHIs were conducted in the United Kingdom (University of Oxford and Imperial College London) and in the Netherlands in during the COVID-19 pandemic (early 2021) without known proven treatment available at that time.

In a CHI study, a *well characterised* strain of an infectious agent is given to *carefully* *selected healthy adult research participants, in* *controlled environment* to better understand how pathogens infect human hosts and cause disease, transmission, and find new ways to prevent and treat infectious diseases. CHIs may be conducted to, better understand immune responses to infection, or to evaluate the efficacy of vaccines and or medicines designed to prevent and treat infectious diseases

As a research design/method, CHIs are relatively new in low and middle income countries, however these have been used for centuries (*date back to 18^th^ and 19^th^*) in high income countries with well-established health systems and research expertise. The CHI methodology has been used for a wide range of diseases, including malaria, influenza, dengue, norovirus, rhinovirus, typhoid, streptococcus, and most recently COVID-19 . Controlled human malaria and influenza infections are the two most frequently practiced. Studies using the controlled human infection method/platform require smaller number of research participants, shorter time frame and provide an early signal of efficacy that may allow for further product optimization as compared to the traditional RCT particularly phase 2 and phase 3.

Although, CHI studies are valuable and have contributed to answering important scientific and public health questions, the concept of intentional infection, directly flouts the principle of non-maleficence and seems at odds with other acceptable ethical principles and standards. There are unique ethical, safety and scientific challenges associated with CHI studies meaning that robust governance and appropriate regulation is essential to their effective use and continued growth. **Below are some links with videos to provide more information about the CHI methodology**

<https://www.youtube.com/watch?v=FcD7kH5mCLk> (CHI Video welcome trust)- What are controlled human infection studies?

<https://www.youtube.com/watch?v=g-DV453M3As> (what are human infection studies) – COVID- 19

<https://www.youtube.com/watch?v=FncT4ki-Uww> (Imperial College London – HIC- VAC) COVID-19 Human infection studies
