# Supplementary material for: “… Infections are not confined in labs…”: Community engagement for Controlled Human Infection Studies: Opinions of researchers, bioethicists and research regulators in Uganda
Source: PLoS One. 2026 Jul 28;21(7):e0353964. doi: 10.1371/journal.pone.0353964 (PMC13411932; doi:10.1371/journal.pone.0353964)
Supplement: S4 Text — (DOCX) [file pone.0353964.s004.docx]

**COMMUNITY ENGAGMENT FOR CONTROLLED HUMAN INFECTION STUDIES**

| Name | Description | Files | References |
| --- | --- | --- | --- |
| 1.0 COMMUNITY ENGAGEMENT | Perceptions on the rationale of community engagement, required time points for engagement and how it should be done | 27 | 254 |
| 1.1 Positive perceptions including opportunities, benefits |  | 13 | 60 |
| Awareness creation about CHIs |  | 4 | 6 |
| Enables design better strategies for recruitment and follow-up |  | 1 | 1 |
| Enhances dissemination of results |  | 3 | 35 |
| Get feedback-learn from or about participant community -Enables filtering information from community to address gaps like myths |  | 4 | 4 |
| Good working relationship with community |  | 2 | 3 |
| Improves acceptability of study |  | 4 | 5 |
| To involve leadership of the community on time sometimes we have to bring in the OC station, |  | 3 | 3 |
| To take precaution from infection... |  | 1 | 1 |
| 2.2 Negative perceptions including barriers of CE |  | 10 | 27 |
| Media as key stakeholder-can distort information |  | 9 | 26 |
| 2.3 When should community engagement be done |  | 11 | 12 |
| A Necessity-From idea inception |  | 10 | 11 |
| Consenting to dissemination |  | 1 | 1 |
| 2.4 Avenues or means for CE |  | 21 | 71 |
| CAB |  | 3 | 3 |
| community events |  | 1 | 1 |
| Community group formation |  | 1 | 1 |
| Community meetings |  | 1 | 1 |
| Community Public address systems |  | 1 | 1 |
| Community sensitisation |  | 1 | 1 |
| community surveys |  | 1 | 1 |
| Dialogue |  | 4 | 4 |
| Government partners |  | 2 | 2 |
| Mass media; Radio show, TVs |  | 10 | 31 |
| Social media |  | 1 | 1 |
| Parliamentary talks |  | 1 | 1 |
| Social media |  | 8 | 11 |
| Through opinion leaders |  | 4 | 4 |
| Through sports activities e.g football match |  | 1 | 1 |
| Train journalists |  | 1 | 1 |
| With scientists in the field-expert |  | 2 | 3 |
| Workshops |  | 3 | 3 |
| 2.5 Stakeholders to engage |  | 19 | 81 |
| Community advisory board-CAB |  | 4 | 4 |
| Community sensitization |  | 1 | 1 |
| CSO-NGOs |  | 1 | 1 |
| DHOs |  | 1 | 1 |
| DSMB- the data safety management boards |  | 4 | 5 |
| Ethical person |  | 1 | 1 |
| Everyone ... |  | 8 | 10 |
| Government |  | 5 | 6 |
| Health workers in the facilities |  | 3 | 4 |
| A Necessity-From idea inception |  | 5 | 6 |
| Community advisory board-CAB |  | 2 | 2 |
| Everyone ... |  | 7 | 9 |
| Health workers in the facilities |  | 1 | 1 |
| Lawyers for legal policy |  | 1 | 1 |
| Local community leaders |  | 2 | 2 |
| Potential participants |  | 1 | 1 |
| Regulatory-RECs, UNCST |  | 2 | 2 |
| The academia |  | 1 | 1 |
| VHTs |  | 1 | 1 |
| Influencial people in the community |  | 1 | 1 |
| International community |  | 1 | 1 |
| Lawyers for legal policy |  | 3 | 5 |
| Local community leaders |  | 7 | 11 |
| MOH |  | 2 | 2 |
| NDA |  | 1 | 1 |
| NIH |  | 1 | 1 |
| PI |  | 1 | 1 |
| Police |  | 1 | 1 |
| Potential participants |  | 3 | 3 |
| Priest |  | 1 | 1 |
| REC |  | 2 | 2 |
| Regulatory-RECs, UNCST |  | 5 | 7 |
| Religious leaders |  | 1 | 1 |
| Researchers |  | 1 | 1 |
| social scientists |  | 1 | 1 |
| The academia |  | 1 | 1 |
| VHTs |  | 2 | 2 |
| Person responsible for community engagement |  | 1 | 1 |
